# Perceived Acceptability of Technology Modalities for the Provision of Universal Child and Family Health Nursing Support in the First 6-8 Months After Birth: Cross-Sectional Study

**DOI:** 10.2196/59191

**Published:** 2024-09-24

**Authors:** Tessa Delaney, Jacklyn K Jackson, Alison L Brown, Christophe Lecathelinais, Luke Wolfenden, Nayerra Hudson, Sarah Young, Daniel Groombridge, Jessica Pinfold, Paul David Craven, Sinead Redman, John Wiggers, Melanie Kingsland, Margaret Hayes, Rachel Sutherland

**Affiliations:** 1 Hunter New England Local Health District New Lambton Australia; 2 School of Medicine and Public Health The University of Newcastle NSW Callaghan Australia; 3 Hunter Medical Research Institute Newcastle Australia

**Keywords:** maternal, postnatal, postpartum, acceptability, technology, digital health, first 2000 days, child health, experience, experiences, attitude, attitudes, opinion, opinion, perception, perceptions, perspective, perspectives, acceptance, cross sectional, survey, surveys, questionnaire, questionnaires, pediatric, pediatrics, infant, infants, infancy, baby, babies, neonate, neonates, neonatal, newborn, newborns, nurse, nurses, nursing

## Abstract

**Background:**

Child and Family Health Nursing (CFHN) services provide universal care to families during the first 2000 days (conception: 5 years) to support optimal health and developmental outcomes of children in New South Wales, Australia. The use of technology represents a promising means to encourage family engagement with CFHN services and enable universal access to evidenced-based age and stage information. Currently, there is little evidence exploring the acceptability of various models of technology-based support provided during the first 2000 days, as well as the maternal characteristics that may influence this.

**Objective:**

This study aims to describe (1) the acceptability of technology-based models of CFHN support to families in the first 6 months, and (2) the association between the acceptability of technology-based support and maternal characteristics.

**Methods:**

A cross-sectional survey was undertaken between September and November 2021 with women who were 6-8 months post partum within the Hunter New England Local Health District of New South Wales, Australia. Survey questions collected information on maternal demographics and pregnancy characteristics, perceived stress, access to CFHN services, as well as preferences and acceptability of technology-based support. Descriptive statistics were used to describe the characteristics of the sample, the proportion of women accessing CFHN services, maternal acceptability of technology-based support from CFHN services, and the appropriateness of timing of support. Multivariable logistic regression models were conducted to assess the association between maternal characteristics and the acceptability of technology-based CFHN support.

**Results:**

A total of 365 women participated in the study, most were 25 to 34 years old (n=242, 68%), had completed tertiary level education or higher (n=250, 71%), and were employed or on maternity leave (n=280, 78%). Almost all (n=305, 89%) women reported accessing CFHN services in the first 6 months following their child’s birth. The majority of women (n=282-315, 82%-92%) “strongly agreed or agreed” that receiving information from CFHN via technology would be acceptable, and most (n=308) women “strongly agreed or agreed” with being provided information on a variety of relevant health topics. Acceptability of receiving information via websites was significantly associated with maternal employment status (*P*=.01). The acceptability of receiving support via telephone and email was significantly associated with maternal education level (adjusted odds ratio 2.64, 95% CI 1.07-6.51; *P*=.03 and adjusted odds ratio 2.90, 95% CI 1.20-7.00; *P*=.02, respectively). Maternal age was also associated with the acceptability of email support (*P*=.04).

**Conclusions:**

Technology-based CFHN support is generally acceptable to mothers. Maternal characteristics, including employment status, education level, and age, were found to modify the acceptability of specific technology modalities. The findings of this research should be considered when designing technology-based solutions to providing universal age and stage child health and developmental support for families during the first 2000 days.

## Introduction

The first 2000 days of a child’s life (conception: 5 years) is a critical time for physical, cognitive, social, and emotional development [1]. Routine health care services or interventions provided in early life have been shown to be protective of poor health outcomes and improve early life experiences, such as learning outcomes, mental well-being, and relationships, as well as healthy growth and development [2,3]. Future health outcomes for children are influenced by these early life experiences and exposures, and subsequently the cumulative effects of positive or negative later life experiences [3].

Given the first 2000 days are a critical period of child health and development, the World Health Organization and governments internationally have released policy frameworks and guidelines that outline strategies and objectives to support the health and development of children during the first 2000 days [4,5]. One example is the First 2000 Days implementation strategy, a government framework that has been released in New South Wales (NSW), Australia [1,6]. A key objective within this framework is that NSW Health provides universal access to child and family health care via Child and Family Health Nursing (CFHN) services. CFHN services support the health and development of children from birth to 5 years across the state through a family centered approach. Services routinely offered by CFHN services include universal health home visits in the first month of a child’s life, postnatal care, immunizations, child health, and developmental checks (through the Personal Health Record or “Blue Book”), feeding support and maternal psychosocial assessments and screening [1]. Despite the availability of this comprehensive service, only half of the 100,000 families of children born each year within NSW continue to access CFHN services within the first year [7], limiting the capacity of CFHN services to provide ongoing and universal health care to families consistent with best practice guidelines.

The delivery of health care services via technology represents a promising way to increase family engagement with CFHN services and provide universal access to evidence-based information consistent with recommendations. In Australia, the COVID-19 pandemic resulted in the widespread adoption of digital technologies to deliver health care services [8-10] including care routinely offered by CFHN services. For example, CFHN services at a local and state level currently use technology, such as telehealth, email, and social media, to support the delivery of care to women and families across NSW [11,12]. While emerging evidence supports the effectiveness and acceptability of nurse-delivered telehealth consultations [11,12], to our knowledge, there is little evidence of the acceptability of other models of technology-based support provided to families during the first 2000 days.

While characteristics, such as age, education, computer literacy, ethnicity, employment, socioeconomic position, and gender, have been associated with the uptake and acceptability of digital health interventions more broadly [13,14], there is limited evidence that comprehensively examines associations between maternal characteristics and the acceptability of technology-based CFHN services, which may be important for designing services that are tailored to individual needs [15]. Given the current evidence gaps, this study aimed to describe (1) the acceptability of technology-based models of CFHN support to families in the first 6 months, by differing health topics, as well as preferences for timing of information receipt; and (2) the association between the acceptability of technology-based support by maternal characteristics hypothesized to influence adoption of technology-based interventions.

## Methods

### Ethical Considerations

This study is reported in accordance with the STROBE (Strengthening the Reporting of Observational Studies in Epidemiology) guidelines [16]. Ethical approval to undertake the study was obtained from the Hunter New England (HNE) Human Research Ethics Committee (16/11/16/4.07), Aboriginal Health and Medical Research Council (1236/16), and the University of Newcastle Human Research Ethics Committee (H-2017-0032). This research was conducted in compliance with informed consent guidelines and adhered to national law and regulations regarding the protection of personal information, privacy, and human rights.

### Study Design and Setting

A cross-sectional survey conducted via computer-assisted telephone interview (CATI) was undertaken between September and November 2021 with women who were 6-8 months post partum within the HNE Local Health District of NSW, Australia. The HNE area is a socioeconomically and geographically diverse region covering approximately 130,000 square kilometers, encompassing major metropolitan, regional, and remote locations [17]. In 2020, there were 10,377 births in the HNE region, accounting for 11.2% of births in NSW [18].

### Context

In NSW, CFHN services provide public health care to women and their families in the child’s first 5 years of life. Health professionals that attend to these services include child and family health nurses, registered midwives, doctors, allied health workers, Aboriginal health workers, and practitioners [19]. Across NSW, there are approximately 417 CFHN services, and approximately 16.3% of CFHN services are located in the HNE Local Health District [19]. The provision of CFHN services may include but not be limited to health home visits, breastfeeding or infant feeding education and support, maternal and child routine screening (ie, maternal psychosocial screening and child vision and hearing screening), child health checks, immunizations, contraception, mental health, and parenting education [20].

### Sample and Recruitment

Women who were 26-37 weeks (6-8 months) post partum, had received antenatal care from public maternity services in the HNE region (responsible for the provision of antenatal care to approximately 70% of women across the district) [17,18] and had previously participated in an antenatal survey while pregnant and agreed to be contacted for future surveys were eligible to participate in the study [21,22]. As per eligibility criteria for the initial antenatal survey, women were ineligible if they were younger than 18 years of age, had an unfortunate pregnancy-related outcome, including stillbirth or death of child, or were not proficient in English preventing them from undertaking the survey unaided.

### Recruitment Procedure

All women in the sampling frame (N=713) were invited to participate in the CATI via a mailed written information statement. The written information statement included an outline of the purpose of the survey and a toll-free number to opt-out or decline survey participation. Electronic medical record data (ie, child’s date of birth and live birth) and previous antenatal survey data (ie, consent to be contacted again) were used to generate a weekly sample of eligible women over an 8-week period. A weekly sample of 100 women were approached to participate in the study for the first 6 weeks, with 68 and 47 women approached in the final 2 weeks of recruitment, respectively. Participants were approached in descending order of their child’s date of birth (ie, parents of older babies were approached first).

### Recruitment Procedure for Non-Aboriginal Women

One week after information statements were mailed, non-Aboriginal women were contacted via telephone and invited by a female interviewer to participate in the survey through a CATI. Women received up to 10 phone attempts over a two-week period to invite study participation. As per formal ethics approval, verbal consent to participate in the study was sought from women during the CATI. Women who declined to participate during the CATI were offered the opportunity to complete the web-based survey. Women who opted to complete the web-based survey were sent an individual survey link to their mobile number or email address. Prior to accessing the web-based survey, women were reminded, on the survey’s display screen, that participation was voluntary and that it was possible to decline the survey at any point. Women’s consent into the study and survey completion status (both via the CATI and web-based) were saved into a central survey database held by the research team.

### Recruitment Procedure for Aboriginal and Torres Strait Islander Women

As per advice received through local cultural consultation processes, women of Aboriginal or Torres Strait Islander origin and/or women who attended or were enrolled to attend an Aboriginal Maternal and Infant Health Service as identified via medical record data were sent an SMS text message after the mail out of the information statement. The SMS text message offered women one of three options as follows: (1) to complete the survey via CATI; (2) to complete the web-based survey; or (3) to decline participation. As per the procedures described above, women who opted to complete the web-based survey were sent an individual survey link to their mobile number which was active for 2 weeks. Women who opted to complete the survey via telephone or did not reply to the SMS text message within 5 days were contacted via telephone and invited to participate in the study by a female interviewer. Women who declined participation via SMS text message were recorded in the central survey database. All women who opted to complete either the telephone or web-based survey were given the opportunity to identify as Aboriginal or Torres Strait Islander or both (regardless of their previous medical record or antenatal survey data). As per ethics and local consultation processes, women who identified as Aboriginal and/or Torres Strait Islander during the CATI were offered the choice of undertaking the survey with a female Aboriginal interviewer.

### Data Collection Procedures

Both the CATI and web-based surveys were developed in REDCap (Research Electronic Data Capture; Vanderbilt University) electronic data capture tools [23]. Survey consent and responses were also stored in REDCap which acted as the central survey database. All survey items were developed and based on local, state, and national health surveys with postpartum women [24,25] and surveys conducted in similar health settings (ie, antenatal services) to assess self-reported acceptability and care by the health service [22]. The surveys were reviewed by child and family health nurses, dietitians, Aboriginal health care workers, and end users (mothers and Aboriginal and Torres Strait Islander women) and pilot-tested prior to use.

### Outcome Measures

Women were asked the following questions in this survey: Aboriginal and/or Torres Strait Islander origin; country of birth; residing postcode, current employment status (full-time, part-time, casual, paid or unpaid maternity leave, unemployed, home duties, student, retired, full-time carer, unable to work due to health problems); timing of their return to work after birth (in months); the child’s date of birth. The survey items were adapted from previous surveys with postpartum women [25] and the Australian Infant Feeding Study [24]. Maternal education status, first or subsequent pregnancy status, and maternal age were not collected in this survey as they were previously collected via the initial antenatal survey with women or medical record data [22]. Participants were asked about their perceived stress via the “perceived stress scale” [26]. The tool is a validated 10-item scale that asks participants to rate their feelings and thoughts in the last month on a 5-point Likert scale from 0 (never) to 4 (very often). For example, the first item asks “In the last month, how often have you been upset because of something that happened unexpectedly?”

Participants were asked about whether they had accessed CFHN services in the first six months since the birth of their baby (yes or no) and the location of the visit (home or clinic).

### Preferences and Acceptability of Technology-Based Support for Child and Family Health Services

Questions around the acceptability of perceived models of care were developed using a 5-point Likert scale (strongly agree to disagree) and were informed using previous surveys with women attending antenatal services [22]. To assess the perceived acceptability for various technology-based CFHN service provision models, mothers were asked: “Can you tell me if you strongly agree, agree, neither agree or disagree, disagree or strongly disagree with receiving information from the health service to support the health of you and your baby for each of these models of technology?” Where five digital delivery models (accessible within current NSW health care systems) were listed (1) SMS text message, (2) website, (3) telehealth services, (4) phone, (5) email, and women were prompted to indicate their acceptability for each option listed. To assess preferences for health-related topics, mothers were asked “Please tell me whether you believe it would be okay to receive support and advice on these topics via technology-based services. You can respond with strongly agree, agree, neither agree or disagree, disagree or strongly disagree.” A list of 11 health-related topics (informed by key Blue Book topics aligned with focus areas of CFHN service provision) were given as options for this question including (1) breastfeeding or bottle feeding, (2) growth checks and immunization reminders, (3) introduction to solids (including timing, portion and type of foods), (4) fussy eating, (5) sleep and settling, (6) age and stage developmental milestones, (7) healthy growth, (8) healthy eating, (9) mental health, (10) social support, (11) parent groups or networks, and women were able to select all response options that applied. Preferences for the timing of the receipt of technology-based support were assessed by asking: “In addition to usual care, if the health service was to provide families with information and support via technology about feeding your baby, when would be the ideal time to receive this information.” Women were instructed to select all response options that applied, including (1) 1st trimester, (2) 2nd trimester, (3) 3rd trimester, (4) 0-6 months after birth, and (5) 7-12 months after birth.

### Characteristics Associated With Perceived Acceptability of Technology-Based CFHN Models of Care

We explored if there were any differences in acceptability by technology-based models of CFHN support by characteristics hypothesized to influence acceptability and use of digital health interventions [13] such as maternal age, maternal education, socioeconomic area, geographical remoteness, current employment status, first pregnancy, Aboriginal or Torres Strait Islander origin, perceived stress, and CFHN service access (in first 6 months post birth).

### Data Analysis

All data were analyzed using the statistical software package SAS (version 9.3; SAS Institute). Descriptive statistics were used to describe the (1) characteristics of the sample, (2) the proportion of women accessing child and family health services in the first 6 months, (3) acceptability of receipt of technology-based support from child and family health services, and (4) appropriate timing of information receipt. Data regarding the characteristics of the sample are presented categorically. Maternal age and timing of return to work after birth were trichotomized where maternal age was categorized as “18-24 years,” “25-34 years,” and “≥35 years” and women’s timing of return to work after birth was categorized as “0-3 months,” “4-6 months,” and “>6 months.” Condensed response categories were created for (1) Aboriginal or Torres Strait Islander Origin (“Aboriginal and/or Torres Strait Islander” or “Neither Aboriginal or Torres Strait Islander” and “don’t know”), (2) maternal education (“high school or less” or “tertiary education or more”), and women’s current employment status including “employed” (full-time, part-time, or casual); “maternity leave” (paid or unpaid) or “unemployed” (home duties, unemployed, retirees, or full-time carers). Women’s residential postcode was used to determine socioeconomic areas using the 2016 socioeconomic indexes for areas [27], which were dichotomized at the median into areas of “most disadvantage” or “least disadvantage.” Women’s residential postcode was also used to determine geographical remoteness (“major cities” or “regional or remote”) using the Access/Remoteness Index of Australia [28]. Women’s perceived stress was determined using the 10-item perceived stress scale [26] where scores were assigned ranging from 0 to 4 for each respective question. The score for each question was then summed to provide a total score of 40. “Low stress” was defined as a score ranging from 0 to 13, “moderate stress” was defined as a score ranging from 14 to 26, and “high stress” was defined as a score of 27 to 40. Child age at the time of survey completion is presented continuously (mean, SD). Women’s reported acceptability of CFHN via varying models of technology was dichotomized into “acceptable” (agreed or strongly agreed) and “not acceptable” (strongly disagree, disagree, or neutral).

Logistic regression models were created to assess associations between maternal characteristics [ (1) Aboriginal and/or Torres Strait Islander status, (2) age, (3) education, (4) socioeconomic status, (5) geographical remoteness, (6) employment status, (7) first pregnancy, (8) perceived stress, and (9) use of CFHN services] and a measure of the acceptability (agree or strongly agree) of technology. Separate logistic regression models were undertaken for each of the five technology models [(1) website, (2) telehealth, (3) telephone, (4) SMS text message, and (5) email], exploring 45 crude logistic regression models in total. Both crude (unadjusted) and models adjusted for all 9 participant characteristics are presented.

In the instance where regression models were unable to produce an odds ratio (OR; if 100% of the group were in one comparison arm), a Haldane-Anscombe correction [29] was applied, whereby the data was weighted in order to add 0.5 to each cell frequency. Statistical significance was set at *P*<.05.

## Results

### Characteristics of Sample

A total of 356 (50% response rate) women participated in the study. While most of the characteristics between the consenting and nonconsenting sample were similar, self-reported Aboriginal and/or Torres Strait Islander status was significantly (*P*=.03) higher within the nonconsenting sample (n=36, 11.6%) compared with the consenting sample (n=23, 6.6%). The majority (n=315, 88%) of consenting study participants were born in Australia with 57% (n=202) of participants residing in major cities and 43% (n=154) of participants in regional or remote locations. Aboriginal and/or Torres Strait Islander women represented 7% (n=23) of the sample. Most of the women were between the ages of 25 and 34 years old (n=242, 68%), had completed tertiary level education or higher (n=250, 71%), and were on maternity leave (n=140, 39%). Of those that were currently employed (n=140), just over half (n=79, 56%) returned to work between 4 and 6 months after birth. The majority of women (n=205, 58%) had perceived stress scores rated as “low” at the time of the survey. The full characteristics of the sample are presented in Table 1.

**Table 1:** Characteristics of women who participated in the survey.

Characteristics		Value (N=356)
**Aboriginal and/or Torres Strait Islander^a^, n (%)**		
	Yes	23 (7)
	No or don’t know	325 (93)
**Maternal age (in years), n (%)**		
	18-24	45 (13)
	25-34	242 (68)
	≥35	69 (19)
**Maternal education^a^, n (%)**		
	High school or less	100 (29)
	Tertiary education or more	250 (71)
**Country of birth, n (%)**		
	Australia	314 (88)
	United Kingdom	4 (1)
	New Zealand	7 (2)
	India	6 (2)
	Other	24 (7)
**Socioeconomic area^b^, n (%)**		
	Most disadvantaged	222 (62)
	Least disadvantaged	134 (38)
**Remoteness^c^, n (%)**		
	Major Cities	202 (57)
	Regional or remote	154 (43)
**Employment status at the time of the survey**		
	Employed	140 (39)
	Maternity leave (paid or unpaid)	140 (39)
	Unemployed	76 (21)
**Return to work after birth (in months; n=140), n (%)**		
	0-3	31 (22)
	4-6	79 (56)
	>6	30 (21)
**First pregnancy, n (%)**		
	Yes	143 (41)
	No or don’t know	208 (59)
Age of baby (in weeks), mean, SD		31.2 (3.1)
**Perceived stress^a^, n (%)**		
	Low stress	205 (58)
	Moderate stress	132 (37)
	High stress	18 (5)

^a^There were missing data for this survey item and the denominator does not total 356 (ie, the women did not respond to the item or skipped the question).

^b^Defined by residential postcode using the 2016 Socioeconomic Indexes for Areas.

^c^Defined by residential postcode using the Access/Remoteness Index of Australia.

A total of 89% (n=305) of women reported accessing CFHN services in the first 6 months following their child’s birth, with 86% (n=262) of visits occurring in the home and 58% (n=177) of women having visited a clinic.

### Preferences and Acceptability of Technology-Based Support From Child and Family Health Services

As shown in Table 2, the majority of women “strongly agreed or agreed” that receiving information from the health service via technology would be acceptable (range: 82%-92%; Cronbach α=0.66), with “website” being rated as the most accepted (n=315, 92%). Most women “strongly agreed or agreed” with being provided with information on all health topics via technology (range: 90%-98%). The most accepted topics were “growth checks and immunization reminders” (n=335, 98%), “healthy eating” (n=333, 97%), and “introduction to solids” (n=331, 97%). Women reported a preference to receive information about feeding their baby in the 3rd trimester of pregnancy (n=173, 50%) or 0-6 months after birth (n=201, 59%).

**Table 2:** Women who agree or strongly agree to receipt of CFHN^a^ support via technology-based services during the first 6 months (in addition to usual CFHN care), and timing preferences for provision of support.

Variable		Value (N=343), n (%)
**Mode of technology support rated as “acceptable”^b^**		
	Website	315 (92)
	Telehealth	306 (89)
	Telephone	295 (86)
	Email	287 (84)
	SMS text message	282 (82)
**Acceptability of health topics delivered via technology^b^**		
	Growth checks and immunization reminders	335 (98)
	Healthy eating	333 (97)
	Introduction to solids (timing, portion, and types of foods)	331 (97)
	Breastfeeding or bottle-feeding	323 (94)
	Sleep and settling	322 (94)
	Healthy growth	323 (94)
	Mental health	322 (94)
	Fussy eating	317 (92)
	Social support	316 (92)
	Age and development milestones	314 (92)
	Parent groups or networks	308 (90)
**Timing of information receival^c^**		
	1st Trimester of pregnancy	34 (10)
	2nd Trimester of pregnancy	55 (16)
	3rd trimester of pregnancy	173 (50)
	0-6 months after birth	201 (59)
	7-12 months after birth	33 (10)

^a^CFHN: Child and Family Health Nursing.

^b^“Acceptability” was defined as “agree” or “strongly agree” with receipt of technology-based information.

^c^Women were instructed to select all that apply.

### Associations With Perceived Acceptability of Technology-Based CFHN Models of Care

While the acceptability of technology-based CFHN services was high for participants overall, some associations were found between maternal characteristics and the acceptability of support provided by technology-based CFHN services (Tables 3-5 and Tables S1 and S2 in Multimedia Appendix 1). Website acceptability was significantly associated with employment (*P*=.01), where women had higher odds of reporting website acceptability if they were employed (adjusted OR 3.30, 95% CI 1.22-8.91) or on maternity leave (adjusted OR 5.06, 95% CI 1.61-15.91) compared to women who were unemployed. For telephone acceptability, women who had received a “high school education or less” had higher odds of agreeing or strongly agreeing that support provided by telephone would be acceptable compared to women who had received “tertiary education or higher” (adjusted OR 2.64, 95% CI 1.07-6.51; *P*=.03). Additionally, women who reported it was their first pregnancy had lower odds of telephone acceptability compared to those that had a previously reported pregnancy (adjusted OR 0.37, 95% CI 0.18-0.76; *P*=.007). For email acceptability, women who had received “high school education or less” had higher odds of reporting email support as acceptable compared to those who had a “tertiary education or higher” (adjusted OR 2.90, 95% CI 1.20-7.00; *P*=.02). Similarly, women who had accessed a CFHN service in the first 6 months since birth had a higher odds (adjusted OR 2.44, 95% CI 1.07-5.53; *P*=.03) of reporting email support as acceptable compared with those that had not accessed a CFHN service. Women’s age was also associated with email acceptability (*P*=.04), where women aged 25-34 years reported higher acceptability of email support (n=206, 87%) compared to women aged 18-24 years (n=32, 76%) and those 35 years and older (n=49, 75%). There were no significant differences between maternal characteristics and telehealth or SMS text messaging acceptability.

**Table 3:** Association between participant characteristics and those who perceive the website as an acceptable (agreed or strongly agreed) mode of receiving health support (n=315).

Characteristics		Website acceptability, n (%)	Crude analysis				Adjusted analysis		
			OR^a^ (95% CI)	*P* value		OR (95% CI)		*P* value	
**Aboriginal or Torres Strait Islander, or both (n=311)^b^**					.39				.59
	Yes	20 (87)	0.57 (0.16-2.06)			0.69 (0.17-2.74)			
	No	291 (92)	1 (reference)			1 (reference)			
**Age of women (in years)**					.52				.36
	18-24	38 (90)	0.46 (0.10-2.17)			0.34 (0.06-2.04)			
	25-34	215 (91)	0.50 (0.14-1.72)			0.40 (0.11-1.45)			
	35+	62 (95)	1 (reference)			1 (reference)			
**Education (n=310)^b^**					.76				.24
	High school or less	89 (93)	1.15 (0.47-2.82)			1.85 (0.66-5.20)			
	Tertiary or higher	221 (92)	1 (reference)			1 (reference)			
**Socioeconomic area**					.35				.35
	Most disadvantaged	197 (93)	1.45 (0.67-3.15)			1.57 (0.61-4.01)			
	Least disadvantaged	118 (90)	1 (reference)			1 (reference)			
**Remoteness**					.71				.66
	Major cities	180 (90)	0.86 (0.39-1.90)			0.80 (0.31-2.11)			
	Regional or remote	135 (92)	1 (reference)			1 (reference)			
**Employment**					.006^c^				.01^c^
	Employed	127 (93)	3.01 (1.22-7.42)			3.30 (1.22-8.91)			
	Maternity leave	127 (95)	4.51 (1.64-12.44)			5.06 (1.61-15.91)			
	Unemployed	61 (82)	1 (reference)			1 (reference)			
**First pregnancy (n=311)^b^**					.65				.70
	Yes	129 (93)	1.20 (0.53-2.72)			1.20 (0.48-3.01)			
	No or don’t know	182 (91)	1 (reference)			1 (reference)			
**Perceived stress (n=314)^b^**					.68				.89
	Low stress	182 (93)	1.73 (0.36-8.35)			1.10 (0.20-6.06)			
	Moderate stress	117 (91)	1.30 (0.26-6.38)			0.88 (0.16-4.93)			
	High stress	15 (88)	1 (reference)			1 (reference)			
**Used CFHN^d^ services**					.24				.30
	Yes	282 (92)	1.86 (0.66-5.21)			1.78 (0.59-5.37)			
	No or don’t know	33 (87)	1 (reference)			1 (reference)			

^a^OR: odds ratio.

^b^Indicates that some characteristic data is missing for women reporting websites as acceptable (n=315 in total).

^c^Indicates a significant result.

^d^CFHN: Child and Family Health Nursing.

**Table 4:** Association between participant characteristics and those who perceive telephone as an acceptable (agreed or strongly agreed) mode of receiving health support (n=295).

Characteristic			Telephone acceptability, n (%)		Crude analysis					Adjusted analysis				
					OR^a^ (95% CI)		*P* value			OR (95% CI)			*P* value	
**Aboriginal or Torres Strait Islander, or both (n=291)^b^**								.87						.72
	Yes	20 (87)		1.11 (0.32-3.88)					1.27 (0.34-4.73)					
	No	271 (86)		1 (reference)					1 (reference)					
**Age of women (in years)**								.27						.52
	18-24	36 (86)		0.50 (0.14-1.76)					0.76 (0.17-3.35)					
	25-34	199 (84)		0.45 (0.17-1.19)					0.57 (0.20-1.64)					
	35+	60 (92)		1 (reference)					1 (reference)					
**Education (n=291)^b^**								.08						.03^c^
	High school or less	88 (92)		2.06 (0.92-4.59)					2.64 (1.07-6.51)					
	Tertiary or higher	203 (84)		1 (reference)					1 (reference)					
**Socioeconomic area**								.67						.77
	Most disadvantaged	181 (85)		0.87 (0.46-1.65)					1.12 (0.53-2.35)					
	Least disadvantaged	114 (87)		1 (reference)					1 (reference)					
**Remoteness**								.15						.15
	Major cities	174 (88)		1.56 (0.85-2.88)					1.69 (0.82-3.45)					
	Regional or remote	121 (83)		1 (reference)					1 (reference)					
**Employment**								.61						.27
	Employed	114 (84)		0.81 (0.36-1.82)					0.93 (0.39-2.24)					
	Maternity leave	117 (88)		1.14 (0.49-2.66)					1.71 (0.65-4.52)					
	Unemployed	64 (86)		1 (reference)					1 (reference)					
**First pregnancy (n=292)^b^**								.004^c^						.007^c^
	Yes	111 (80)		0.39 (0.21-0.75)					0.37 (0.18-0.76)					
	No or don’t know	181 (91)		1 (reference)					1 (reference)					
**Perceived stress (n=294)^b^**								.85						.66
	Low stress	170 (87)		1.40 (0.38-5.21)					1.84 (0.44-7.64)					
	Moderate stress	110 (85)		1.24 (0.33-4.73)					1.96 (0.46-8.44)					
	High stress	14 (82)		1 (reference)					1 (reference)					
**Used CFHN^d^ services**								.26						.25
	Yes	260 (85)		0.50 (0.15-1.68)					0.48 (0.13-1.69)					
	No or don’t know	35 (92)		1 (reference)					1 (reference)					

^a^OR: odds ratio.

^b^Indicates that some characteristic data is missing for women reporting telephone as acceptable (n=295 in total).

^c^Indicates a significant result.

^d^CFHN: Child and Family Health Nursing.

**Table 5:** Association between participant characteristics and those who perceive email as an acceptable (agreed or strongly agreed) mode of receiving health support (n=287).

Characteristic		Email acceptability, n (%)	Crude analysis				Adjusted analysis			
			OR^a^ (95% CI)	*P* value		OR (95% CI)			*P* value	
**Aboriginal or Torres Strait Islander, or both (n=283)^b^**					.07					.07
	Yes	16 (70)	0.42 (0.16-1.07)			0.39 (0.14-1.07)				
	No	267 (84)	1 (reference)			1 (reference)				
**Age of women (years)**					.03^c^					.04^c^
	18-24	32 (76)	1.04 (0.42-2.59)			0.77 (0.23-2.54)				
	25-34	206 (87)	2.24 (1.13-4.43)			2.06 (0.98-4.33)				
	35+	49 (75)	1 (reference)			1 (reference)				
**Education (n=282)^b^**					.03^c^					.02^c^
	High school or less	87 (91)	2.28 (1.07-4.86)			2.90 (1.20-7.00)				
	Tertiary or higher	195 (81)	1 (reference)			1 (reference)				
**Socioeconomic area**					.05^c^					.13
	Most disadvantaged	184 (87)	1.79 (1.00-3.18)			1.70 (0.85-3.41)				
	Least disadvantaged	103 (79)	1 (reference)			1 (reference)				
**Remoteness**					.26					.76
	Major cities	161 (82)	0.71 (0.39-1.29)			0.89 (0.43-1.85)				
	Regional or remote	126 (86)	1 (reference)			1 (reference)				
**Employment**					.63					.56
	Employed	117 (86)	1.31 (0.61-2.84)			1.38 (0.58-3.27)				
	Maternity leave	109 (82)	0.97 (0.46-2.04)			0.96 (0.40-2.34)				
	Unemployed	61 (82)	1 (reference)			1 (reference)				
**First pregnancy (n=283)^b^**					.91					.76
	Yes	116 (83)	0.97 (0.54-1.74)			0.90 (0.44-1.81)				
	No or don’t know	167 (84)	1 (reference)			1 (reference)				
**Perceived stress (n=286)^b^**					.18					.24
	Low stress	169 (86)	2.61 (0.85-7.99)			2.93 (0.84-10.22)				
	Moderate stress	105 (81)	1.82 (0.59-5.66)			2.43 (0.68-8.61)				
	High stress	12 (71)	1 (reference)			1 (reference)				
**Used CFHN^d^ services**					.03^c^					.03^c^
	Yes	260 (85)	2.35 (1.09-5.08)			2.44 (1.07-5.53)				
	No or don’t know	27 (71)	1 (reference)			1 (reference)				

^a^OR: odds ratio.

^b^Indicates that some characteristic data is missing for women reporting email as acceptable (n=287 in total).

^c^Indicates a significant result.

^d^CFHN: Child and Family Health Nursing.

## Discussion

### Principal Findings

This study describes the acceptability of technology-based models of CFHN support to families in the first 6 months post partum and identifies maternal characteristics that may influence the acceptability or adoption of such technology-based models. Our findings indicate most mothers (90%-98%) would find receiving information on key health topics via technology-based platforms acceptable. While there was high acceptability across a range of maternal characteristics, employment status, education level, and age were significantly associated with maternal acceptability of receiving various types of digital support and should be considered when developing technology-based models of CFHN support or care.

The technology platform that the highest proportion of mothers found acceptable for receiving health information was websites (n=315, 92%), followed by telehealth (n=306, 89%), telephone (n=295, 86%), email (n=287, 84%) and SMS text messaging (n=282, 82%). Given that mothers universally (ie, >80%) reported these technology-based platforms as acceptable, these findings suggest that various models of technology-based support would be suitable for providing families with child health and parenting support. This finding is largely consistent with the wider published literature, indicating that pregnant women and new mothers believe that the use of digital platforms, such as websites or SMS text messages, is a preferable and appealing method for receiving health information due to the convenience of the delivery models [30,31].

Almost all mothers surveyed in this study indicated that they would like to receive information related to growth checks and immunizations (n=335, 98%), healthy eating (n=333, 97%), and the introduction of solids (n=331, 97%). However, at least 90% of mothers indicated an interest in another eight topics including breastfeeding or bottle feeding, sleep and settling, and healthy growth, highlighting that new mothers are interested in accessing information across a broad spectrum of health topics related to their babies. Approximately 50% of mothers indicated a preference for receiving health information during the 3rd trimester and 59% of mothers in the first 6 months after birth. This finding is consistent with previous qualitative research that found mothers were most open to receiving parenting information closer to or after the baby was born, but not while in the hospital [30]. The 3rd trimester and first 6 months after birth when the mother is home with her newborn represents a highly receptive period for providing mothers with supportive, relevant, and reliable child health and parenting information.

Early motherhood represents a period in which women are interested in accessing a wide range of parenting information. Increasingly, the use of digital media sources represents an opportunity for women to access information that is of relevance to them, and at times that is convenient. Specifically, qualitative data has indicated that mothers tend to favor digital media during early motherhood, as they valued that the information was: (1) immediate (ie, quick Google search), (2) regular (ie, regular release of information without their intervention), (3) detailed, (4) entertaining (ie, relatable content or means of alleviating boredom), (5) customized, (6) practical (ie, how to), (7) professional, (8) reassuring, and (9) unbiased (ie, noncommercial) [32]. However, the most acceptable and equitable digital or technology mode for supporting mothers to assess child health and parenting information during this time remains unclear.

To the authors’ knowledge, very few studies have previously examined the association between the acceptability of various technology-based models of CFHN care with maternal characteristics [33,34]. Our findings indicated that websites had higher odds of being acceptable if the mother was employed or on maternity leave. An Australian study conducted in 2010 by Wen et al [35] found that lower-income households and less educated mothers tended to have lower rates of internet access for accessing health information. These findings suggest a possible inequity of websites for providing child health and parenting information, however, the spread of smartphones and internet access over the past decade has likely reduced this potential inequity [35]. In addition, we found higher odds of telephone and email support being acceptable if the mother had received a “high school education or less” compared with women who received a “tertiary education or higher.” Email was also more acceptable if the mother had accessed the CFHNs in the first 6 months following the birth, and if the mother was 25-34 years old. Maternal characteristics were not significantly associated with the acceptability of telehealth or SMS text messaging modalities, suggesting that CFHN support offered through these platforms may be more equitably accessed by mothers, however, further research exploring this topic is timely.

### Limitations

Limitations of this research include the cross-sectional nature of the study and possible selection bias (influenced by a 50% response rate), which may limit the generalizability of these findings. Additionally, the sampling of mothers may have been slightly skewed to include mothers who are more engaged with CFHN services, as 89% (n=305) of mothers in the sample accessed CFHNs in the first 6 months following birth, which is higher than HNE-wide CFHN service attendance (<70%). However, the survey question used to capture this data did not ask if mothers attended all scheduled CFHN service visits in the first 6 months, therefore participant engagement with CFHN services may appear artificially high, as other characteristics of the sample are similar to that of the broader HNE region [36], with approximately 62% of mothers aged between 25 and 34 years and 91.5% from English speaking backgrounds [37]. Although previous research has demonstrated high acceptability and feasibility for delivering health advice to women using mobile apps [38], this research did not explore the acceptability of mobile apps given the well-known technological issues often experienced with the development and upkeep of mobile apps [39]. Rather, this research focused only on technology-based platforms that are already readily available within the health service. As such gaps remain in our understanding of mother acceptability for a range of possible technology platforms for delivering CFHN care, outside of those assessed in this study. Additionally, some of the regression analyses conducted for this study produced wide confidence intervals, suggesting a level of uncertainty in some of the associations and should be considered when interpreting the findings.

### Conclusions

The findings of this study indicate that mothers are interested in using technology to access information related to a variety of child health and development topics, specifically within the first 6 months post partum. Technology-based models of providing this support to mothers, alongside CFHN services were found to be highly acceptable among new mothers, however, maternal characteristics, including employment status, education level, and age, were found to significantly modify maternal acceptability of technologies including websites, telephone, and email. Despite mothers indicating an appetite for receiving age and stage-relevant health and development information via technology-based approaches, future research is warranted to ensure technology-based models of CFHN care are accessed equitably by mothers.

## Appendix

Multimedia Appendix 1 Additional tables.

## Abbreviations

CATI: computer-assisted telephone interview
CFHN: Child and Family Health Nursing
HNE: Hunter New England
NSW: New South Wales
OR: odds ratio
REDCap: Research Electronic Data Capture
STROBE: Strengthening the Reporting of Observational Studies in Epidemiology

## References

[1] (2019). Policy directive: the first 2000 days framework. NSW Ministry of Health.

[2] (2021). Brighter beginnings: the first 2000 days of life. NSW Government.

[3] Moore T, Arefadib N, Deery A, West S, Keyes M (2017). The first thousand days: an evidence paper. Centre for Community Health, Murdoch Children's Research Institute.

[4] (2020). Improving early childhood development. WHO.

[5] (2018). Nurturing care for early childhood development: a framework for helping children survive and thrive to transform health and human potential. Nurturing Care Framework for Early Childhood Development.

[6] (2021). First 2000 days implementation strategy 2020-2025. NSW Ministry of Health.

[7] Celebrating 100 years. NSW Child and Family Health Services.

[8] Asadzadeh A, Kalankesh LR (2021). A scope of mobile health solutions in COVID-19 pandemics. Inform Med Unlocked.

[9] Lee SM, Lee D (2021). Opportunities and challenges for contactless healthcare services in the post-COVID-19 Era. Technol Forecast Soc Change.

[10] Parker J, Robinson J, MugicaCox B, Foy A, Kepu K, HarrisRoxas B (2022). How COVID-19 shaped new models of care for a child and family health nursing service. Aust J Child Fam Health Nurs.

[11] Snoswell CL, Chelberg G, De Guzman KR, Haydon HH, Thomas EE, Caffery LJ, Smith AC (2021). The clinical effectiveness of telehealth: a systematic review of meta-analyses from 2010 to 2019. J Telemed Telecare.

[12] James S, Ashley C, Williams A, Desborough J, Mcinnes S, Calma K, Mursa R, Stephen C, Halcomb EJ (2021). Experiences of Australian primary healthcare nurses in using telehealth during COVID-19: a qualitative study. BMJ Open.

[13] Perski O, Blandford A, West R, Michie S (2017). Conceptualising engagement with digital behaviour change interventions: a systematic review using principles from critical interpretive synthesis. Transl Behav Med.

[14] Perski O, Short CE (2021). Acceptability of digital health interventions: embracing the complexity. Transl Behav Med.

[15] Taki S, Russell CG, Wen LM, Laws RA, Campbell K, Xu H, Denney-Wilson E (2019). Consumer engagement in mobile application (App) interventions focused on supporting infant feeding practices for early prevention of childhood obesity. Front Public Health.

[16] von Elm E, Altman DG, Egger M, Pocock SJ, Gøtzsche PC, Vandenbroucke JP, STROBE Initiative (2008). The strengthening the reporting of observational studies in epidemiology (STROBE) statement: guidelines for reporting observational studies. J Clin Epidemiol.

[17] (2022). Hunter New England. NSW Ministry of Health.

[18] (2020). Centre for Epidemiology and Evidence. HealthStats NSW.

[19] (2021). NSW child and family health services. New South Wales Ministry of Health.

[20] (2022). Having a baby. NSW Ministry of Health.

[21] Kingsland M, Hollis J, Farragher E, Wolfenden L, Campbell K, Pennell C, Reeves P, Tully B, Daly J, Attia J, Oldmeadow C, Hunter M, Murray H, Paolucci F, Foureur M, Rissel C, Gillham K, Wiggers J (2021). An implementation intervention to increase the routine provision of antenatal care addressing gestational weight gain: study protocol for a stepped-wedge cluster trial. Implement Sci Commun.

[22] Doherty E, Wiggers J, Wolfenden L, Anderson AE, Crooks K, Tsang TW, Elliott EJ, Dunlop AJ, Attia J, Dray J, Tully B, Bennett N, Murray H, Azzopardi C, Kingsland M (2019). Antenatal care for alcohol consumption during pregnancy: pregnant women's reported receipt of care and associated characteristics. BMC Pregnancy Childbirth.

[23] Patridge EF, Bardyn TP (2018). Research electronic data capture (REDCap). JMA.

[24] (2010). Australian national infant feeding survey: indicator results, summary—Australian institute of health and welfare. Australian Institute of Health and Welfare, 2010 Australian National Infant Feeding Survey.

[25] Wen LM, Rissel C, Xu H, Taki S, Buchanan L, Bedford K, Phongsavan P, Baur LA (2020). Effects of telephone and short message service support on infant feeding practices, "Tummy Time," and screen time at 6 and 12 months of child age: a 3-group randomized clinical trial. JAMA Pediatr.

[26] Cohen S, Kamarck T, Mermelstein R (1983). A global measure of perceived stress. J Health Soc Behav.

[27] (2016). Socio-economic indexes for areas (SEIFA): technical paper. Australian Bureau of Statistics.

[28] Department of Health and Aged Care, National Key Centre for Social Applications of Geographical Information Systems (2001). Measuring remoteness: accessibility/remoteness index of Australia (ARIA). Occasional Papers: New Series Number 14.

[29] Ruxton GD, Neuhäuser M (2012). Review of alternative approaches to calculation of a confidence interval for the odds ratio of a 2 × 2 contingency table. Methods Ecol Evol.

[30] Gazmararian JA, Dalmida SG, Merino Y, Blake S, Thompson W, Gaydos L (2014). What new mothers need to know: perspectives from women and providers in Georgia. Matern Child Health J.

[31] Ekambareshwar M, Mihrshahi S, Wen LM, Taki S, Bennett G, Baur LA, Rissel C (2018). Facilitators and challenges in recruiting pregnant women to an infant obesity prevention programme delivered via telephone calls or text messages. Trials.

[32] Lupton D (2016). The use and value of digital media for information about pregnancy and early motherhood: a focus group study. BMC Pregnancy Childbirth.

[33] Wallwiener S, Müller M, Doster A, Laserer W, Reck C, Pauluschke-Fröhlich J, Brucker SY, Wallwiener CW, Wallwiener M (2016). Pregnancy eHealth and mHealth: user proportions and characteristics of pregnant women using web-based information sources-a cross-sectional study. Arch Gynecol Obstet.

[34] Greene EM, O'Brien EC, Kennelly MA, O'Brien OA, Lindsay KL, McAuliffe FM (2021). Acceptability of the pregnancy, exercise, and nutrition research study with smartphone app support (PEARS) and the use of mobile health in a mixed lifestyle intervention by pregnant obese and overweight women: secondary analysis of a randomized controlled trial. JMIR Mhealth Uhealth.

[35] Wen LM, Rissel C, Baur LA, Lee E, Simpson JM (2011). Who is NOT likely to access the internet for health information? Findings from first-time mothers in southwest Sydney, Australia. Int J Med Inf.

[36] (2018). Hunter New England. NSW Health.

[37] NSW mothers and babies 2021. Centre for Epidemiology and Evidence.

[38] Nkabane-Nkholongo E, Mpata-Mokgatle M, Jack BW, Julce C, Bickmore T (2024). Usability and acceptability of a conversational agent health education app (Nthabi) for young women in lesotho: quantitative study. JMIR Hum Factors.

[39] Shorey S, Tan TC, Mathews J, Yu CY, Lim SH, Shi L, Ng ED, Chan YH, Law E, Chee C, Chong YS, thilagamangai (2021). Development of a supportive parenting app to improve parent and infant outcomes in the perinatal period: development study. J Med Internet Res.

