# Supplementary material for: Perceived Acceptability of Technology Modalities for the Provision of Universal Child and Family Health Nursing Support in the First 6-8 Months After Birth: Cross-Sectional Study
Source: JMIR Pediatr Parent. 2024 Sep 24;7:e59191. doi: 10.2196/59191 (PMC11462103; doi:10.2196/59191)
Supplement: Multimedia Appendix 1 [file pediatrics_v7i1e59191_app1.docx]

**Table S1.** Association between participant characteristics and those who perceive telehealth as an acceptable (agreed or strongly agreed) (n=306) mode of receiving health support.

| Characteristic | Telehealth acceptability  N (%) | Crude Analysis | | Adjusted Analysis | |
| --- | --- | --- | --- | --- | --- |
|  |  | Odds Ratio (95% CI) | *P* value | Odds Ratio (95% CI) | *P* value |
| **Aboriginal or Torres Strait Islander, or both (n=302)^b^** | | | | | |
| Yes | 23 (100%)^a^ | 6.30  (0.38-105.87) | .20 | 7.75  (0.45-133.28) | .16 |
| No | 279 (88%) | 1 [Reference] |  | 1 [Reference] |  |
| **Age of women (years) (n=306)** | | | | | |
| 18-24 | 35 (83%) | 0.33  (0.09-1.20) | .24 | 0.23  (0.05-1.07) | .17 |
| 25-34 | 210 (89%) | 0.53  (0.18-1.58) |  | 0.51  (0.16-1.58) |  |
| 35+ | 61 (94%) | 1 [Reference] |  | 1 [Reference] |  |
| **Education (n=300)^b^** | | | | | |
| High school or less | 88 (92%) | 1.50  (0.66-3.42) | .33 | 2.21  (0.86-5.67) | .10 |
| Tertiary or higher | 212 (88%) | 1 [Reference] |  | 1 [Reference] |  |
| **Socioeconomic area (n=306)** | | | | | |
| Most disadvantaged | 188 (89%) | 0.86  (0.42-1.76) | .69 | 1.09  (0.48-2.49) | .83 |
| Least disadvantaged | 118 (90%) | 1 [Reference] |  | 1 [Reference] |  |
| **Remoteness (n=306)** | | | | | |
| Major cities | 179 (91%) | 1.49  (0.75-2.95) | .25 | 1.36  (0.61-2.99) | .45 |
| Regional/remote | 127 (87%) | 1 [Reference] |  | 1 [Reference] |  |
| **Employment (n=306)** | | | | | |
| Employed | 123 (90%) | 1.48  (0.61-3.56) | .67 | 1.70  (0.66-4.37) | .53 |
| Maternity leave | 119 (89%) | 1.33  (0.56-3.16) |  | 1.49  (0.57-3.93) |  |
| Unemployed | 64 (86%) | 1 [Reference] |  | 1 [Reference] |  |
| **First pregnancy (n=301)^b^** | | | | | |
| Yes | 121 (87%) | 0.71  (0.36-1.41) | .33 | 0.83  (0.39-1.80) | .64 |
| No/ don’t know | 180 (90%) | 1 [Reference] |  | 1 [Reference] |  |
| **Perceived stress (n=305)^b^** | | | | | |
| Low stress | 173 (88%) | 1.00  (0.22-4.67) | .78 | 0.93  (0.17-5.01) | .81 |
| Moderate stress | 117 (91%) | 1.30  (0.26-6.38) |  | 1.20  (0.21-6.81) |  |
| High stress | 15 (88%) | 1 [Reference] |  | 1 [Reference] |  |
| **Used CFHN services (n=306)** | | | | | |
| Yes | 271 (89%) | 0.68  (0.20-2.34) | .54 | 0.67  (0.19-2.38) | .54 |
| No/ don’t know | 35 (92%) | 1 [Reference] |  | 1 [Reference] |  |

^a^Haldane-Anscombe correction applied, whereby the data was weighted in order to add 0.5 to each cell frequency to generate an odds ratio.

^b^Indicates that some characteristic data is missing for women reporting telehealth as acceptable (n=306 in total).

CFHN, child and family health nurse.

**Table S2.** Association between participant characteristics and those who perceive text message as an acceptable (agreed or strongly agreed) (n=282) mode of receiving health support.

| Characteristic | Text message acceptability N (%) | Crude Analysis | | Adjusted Analysis | |
| --- | --- | --- | --- | --- | --- |
|  |  | Odds Ratio (95% CI) | *P* value | Odds Ratio (95% CI) | *P* value |
| **Aboriginal or Torres Strait Islander, or both (n=278)^a^** | | | | | |
| Yes | 21 (91%) | 2.41  (0.55-10.56) | .24 | 2.79  (0.62-12.58) | .18 |
| No | 257 (81%) | 1 [Reference] |  | 1 [Reference] |  |
| **Age of women (years) (n=282)** | | | | | |
| 18-24 | 34 (81%) | 1.06  (0.40-2.83) | .83 | 1.03  (0.31-3.36) | .89 |
| 25-34 | 196 (83%) | 1.23  (0.61-2.46) |  | 1.17  (0.56-2.46) |  |
| 35+ | 52 (80%) | 1 [Reference] |  | 1 [Reference] |  |
| **Education (n=277)^a^** | | | | | |
| High school or less | 78 (81%) | 0.91  (0.50-1.69) | .77 | 0.95  (0.49-1.87) | .89 |
| Tertiary or higher | 199 (83%) | 1 [Reference] |  | 1 [Reference] |  |
| **Socioeconomic area (n=282)** | | | | | |
| Most disadvantaged | 178 (84%) | 1.36  (0.78-2.38) | .28 | 1.21  (0.64-2.29) | .56 |
| Least disadvantaged | 104 (79%) | 1 [Reference] |  | 1 [Reference] |  |
| **Remoteness (n=282)** | | | | | |
| Major cities | 159 (81%) | 0.78  (0.44-1.38) | .40 | 0.81  (0.42-1.56) | .53 |
| Regional/remote | 123 (84%) | 1 [Reference] |  | 1 [Reference] |  |
| **Employment (n=282)** | | | | | |
| Employed | 116 (85%) | 1.86  (0.91-3.80) | .22 | 1.84  (0.86-3.96) | .29 |
| Maternity leave | 110 (83%) | 1.54  (0.77-3.08) |  | 1.54  (0.70-3.40) |  |
| Unemployed | 56 (76%) | 1 [Reference] |  | 1 [Reference] |  |
| **First pregnancy (n=277)^a^** | | | | | |
| Yes | 113 (81%) | 0.93  (0.53-1.63) | .79 | 0.88  (0.46-1.67) | .69 |
| No/ don’t know | 164 (82%) | 1 [Reference] |  | 1 [Reference] |  |
| **Perceived stress (n=281)^a^** | | | | | |
| Low stress | 163 (83%) | 1.52  (0.47-4.95) | .76 | 1.38  (0.38-4.96) | .87 |
| Moderate stress | 105 (81%) | 1.35  (0.40-4.49) |  | 1.25  (0.34-4.60) |  |
| High stress | 13 (76%) | 1 [Reference] |  | 1 [Reference] |  |
| **Used CFHN services (n=282)** | | | | | |
| Yes | 251 (82%) | 1.05  (0.44-2.51) | .91 | 1.10  (0.45-2.66) | .84 |
| No/ don’t know | 31 (82%) | 1 [Reference] |  | 1 [Reference] |  |

^a^Indicates that some characteristic data is missing for women reporting email as acceptable (n=282 in total).

CFHN, child and family health nurse
